# Metabolomic Analysis of Cauda Epididymal Fluid in Yaks and Cattle

**DOI:** 10.3390/ani15192861

**Published:** 2025-09-30

**Authors:** Gan Yang, Xiaolong Yang, Dongju Liu, Wending Zhou, Anjun Zhou, Yan Xiong, Xianrong Xiong, Wei Fu, Jian Li, Daoliang Lan, Shi Yin

**Affiliations:** 1 College of Animal & Veterinary Sciences, Southwest Minzu University, Chengdu 610041, China; 18288246608@163.com (G.Y.); yxlong0123@163.com (X.Y.); qingjiu001020@163.com (D.L.); zhouwd0123@gmail.com (W.Z.); zhouanjun168@163.com (A.Z.); xiongyan0910@126.com (Y.X.); xianrongxiong@163.com (X.X.); fuwei@swun.edu.cn (W.F.); lijian@swun.cn (J.L.); 2 Key Laboratory of Animal Science of National Ethnic Affairs Commission of China, Southwest Minzu University, Chengdu 610041, China

**Keywords:** epididymal cauda fluid, yak, metabolomics, sperm, male fertility

## Abstract

**Simple Summary:**

Yaks, native to high-altitude regions, hold significant economic and research value, yet exhibit lower reproductive capacity and reduced sperm motility compared to cattle. The epididymis, where sperm maturation occurs, features the cauda region as the primary storage site, with cauda epididymal fluid secreted by epithelial cells forming the microenvironment for sperm maturation. This study used liquid chromatography-mass spectrometry (LC-MS/MS)-based metabolomics to analyze and compare cauda epididymal fluid components between yaks and cattle. We found that under positive and negative ion detection modes, 1098 and 1297 metabolites were identified, respectively, with the most abundant categories being organic acids and derivatives, organoheterocyclic compounds, and lipids and lipid-like molecules. Compared with cattle, yak cauda epididymal fluid showed 79 significantly upregulated and 212 downregulated metabolites in the positive ion mode, and 110 upregulated and 230 downregulated metabolites in the negative ion mode. Among the differentially expressed metabolites across both species, a total of 14 metabolites positively correlated with sperm motility or functional maturation while four negatively associated with them. Our results establish a valuable metabolomic reference dataset for yak reproductive biology and provide new insights into the molecular regulation of sperm function.

**Abstract:**

The epididymis is crucial for sperm maturation, with its caudal region storing mature sperm. Yaks show poorer sperm motility and higher abnormality rates than cattle, but the metabolic mechanisms remain unknown. This study compared cauda epididymal fluid metabolites between six yaks and six cattle using untargeted metabolomics. A total of 1098 and 1297 metabolites types annotated by the Human Metabolome Database were identified in yak and cattle cauda epididymal fluid, respectively, using positive and negative ion modes. The Yak cauda epididymal fluid exhibited distinct metabolic profiles compared with cattle. A total of 79 metabolites were upregulated and 212 were downregulated in the positive ion mode, while 110 were upregulated and 230 were downregulated in the negative ion mode. Among these, 14 metabolites were reported to promote sperm quality, function or metabolism by reducing oxidative stress, blocking premature sperm capacitation and spontaneous acrosome reaction, enhancing mitochondrial energy metabolism or facilitating flagellar motility in cattle or other species. Four were reported to impair the quality or function of sperm via increasing the DNA methylation, inhibiting spermatozoa motility, upregulating the ROS levels and diminishing sperm motility. Taken together, this study established a valuable metabolomic reference dataset for yak reproductive biology and provided new insights into the molecular regulation of sperm function.

## 1. Introduction

The maturation of spermatozoa is closely associated with the epididymis, an organ composed of highly convoluted, narrow ducts. Anatomically, the epididymis is divided into three regions: caput (head), corpus (body), and cauda (tail). The luminal microenvironment, jointly formed by epididymal epithelial cells and their secreted epididymal fluid, plays an indispensable role in sperm transport, maturation, and storage [1,2,3,4,5,6]. The rhythmic contraction of smooth muscle and the directional beating of cilia within the epididymal ductal epithelial cells facilitate spermatozoa transport toward the vas deferens. Besides, the epididymis absorbs rete testis fluid secreted by Sertoli cells, thereby preventing it from impeding sperm transit [7,8,9]. The luminal fluid comprises various components, including proteins, RNAs, inorganic ions, and small organic molecules [10,11,12]. As spermatozoa traverse the epididymis, they interact with key proteins and small molecular compounds in the luminal fluid, undergoing a series of changes including nuclear condensation, alterations in plasma membrane composition, cytoskeletal restructuring, and modifications in protein and non-coding RNA cargoes. By the time sperm exit the epididymis, the majority have acquired both progressive motility and the capacity to fertilize oocytes [13,14,15,16].

Prior to ejaculation, the cauda epididymis serves as a critical reservoir for mature spermatozoa, storing 50% to 80% of the sperm within the epididymal lumen [7,17]. Compared to other epididymal regions, the cauda fluid has higher osmotic pressure, promoting sperm dehydration, tight nuclear condensation, and genetic stability [7,17]. Its low oxygen content suppresses aerobic metabolism, reduces energy consumption, and extends survival [18,19]. The acidic environment also inhibits sperm motility [20,21]. During ejaculation, interactions between cauda fluid proteins and accessory gland secretions may influence sperm function. The unique physicochemical properties and functions of the cauda fluid directly protect the structural and functional integrity of sperm [22,23] Accordingly, an in-depth investigation of this microenvironment is crucial for elucidating the regulatory mechanisms underlying sperm maturation and fertilization. Furthermore, the epididymal fluid has emerged as a valuable source of biomarkers for fertility assessment. Several epididymal-derived molecules, such as specific proteins and metabolites, are currently under investigation for their potential clinical use in diagnosing male infertility in both humans and animals [24,25,26,27]. Identifying such biomarkers could not only enhance our understanding of sperm maturation defects but also lead to practical diagnostic tools and therapeutic strategies for improving reproductive outcomes.

Metabolomics focuses on the qualitative and quantitative analysis of all small-molecule metabolites (e.g., amino acids, organic acids, and sugars) within organisms. In recent years, metabolomics has been widely applied to study the composition of cauda epididymal fluid in single species, such as humans and mice [6,28,29]. However, cross-species comparative metabolomics of epididymal fluid has not been reported. The yak is a native livestock species mainly distributed in the Qinghai–Tibet Plateau and surrounding high-altitude regions, possessing significant scientific research and economic value. Compared with cattle, male yaks under high-altitude conditions exhibit distinct sexual maturity patterns, typically producing mature sperm at 2–3 years of age (compared to 1–1.5 years in cattle) [9,30,31]. This developmental difference may reflect metabolic adaptation strategies to extreme environments. Existing studies have reported lower sperm motility, higher abnormality rates, and poorer post-thaw recovery in the yak frozen semen [32]. We hypothesize that significant metabolic differences exist in the cauda epididymal fluid between yaks and cattle, reflecting distinct physiological adaptations to their respective environments, which may be associated with the species-specific variation in sperm maturation kinetics and functional characteristics. In this study, we applied an untargeted metabolomics approach to compare the epididymal fluid between yaks and cattle, aiming to establish a reference metabolomic dataset for future reproductive research and to elucidate the regulatory mechanisms of sperm function in high-altitude environments. The findings may provide potential targets for enhancing yak reproductive efficiency and practical applications in livestock breeding.

## 2. Materials and Methods

### 2.1. Sample Collection

Samples were obtained from a slaughterhouse in Qingbaijiang District, Chengdu, Sichuan Province. Six healthy male Maiwa yaks and six healthy male cattle (a crossbreed of Simmental cattle and Sichuan local yellow cattle), aged 3–4 years with comparable body weights (mean ± SEM: yak—385 ± 67 kg; cattle—425 ± 50 kg). The animals were raised under typical local extensive grazing conditions based on natural pasture and supplemented with concentrate during winter to keep a BCS > 2.5. In addition, general management considered veterinary clinical assessment and routine vaccination and parasite control programs for endemic diseases. The average ambient temperature was 18.6 ± 4.3 °C and relative humidity was 75 ± 11% during the sampling period. All animals were slaughtered on the same day, were selected (*n* = 6 for each species). Epididymal caudae were dissected under laminar flow, rinsed with a PBS, and placed in a dish containing 2 mL PBS. After making 3–4 incisions (~1 cm), the luminal fluid was expressed gently with forceps. Following 5-min incubation at ambient temperature, the fluid was aspirated and centrifuged at 1500× *g* for 10 min at 4 °C. The supernatant was further centrifuged at 12,000× *g* for 10 min at 4 °C, aliquoted, and stored at −80 °C for metabolomic analysis (*n* = 6 per species). All procedures were approved by the Animal Care and Ethics Committee of Southwest Minzu University (SWU-202501136).

### 2.2. Metabolite Extraction and Quality Control

Aliquots (100 μL) of the samples were transferred to microcentrifuge tubes and resuspended in pre-chilled 80% methanol (Shanghai Macklin Biochemical Co., Ltd. M813903-4 × 4L, Shanghai, China) via vigorous vortex mixing. The mixtures were then incubated on ice for 5 min and centrifuged at 15,000× *g* and 4 °C for 20 min. Then 80 μL of the supernatant was diluted with 40 μL of LC-MS grade water to achieve a final methanol concentration of 53%. The diluted samples were subsequently transferred to fresh microcentrifuge tubes and centrifuged again at 15,000× *g* and 4 °C for 20 min. Finally, the resulting supernatant was subjected to LC-MS/MS analysis [33,34].

To account for potential technical variability and ensure analytical robustness, all samples were randomized prior to the metabolite extraction process. Additionally, a pooled quality-controlled (QC) sample, created by combining equal aliquots from all samples, was analyzed alongside the experimental samples to monitor instrument performance and reproducibility throughout the acquisition sequence.

### 2.3. The UHPLC-MS/MS Analysis

UHPLC-MS/MS analyses were conducted at Novogene (Beijing, China) using a Vanquish UHPLC system (Thermo Fisher Scientific, Germering, Germany) coupled to either an Orbitrap Q Exactive™ HF or HF-X mass spectrometer (Thermo Fisher Scientific, Germering, Germany). Samples were separated on a Hypersil Gold column (with a 100 mm length, 2.1 mm internal diameter, and 1.9 μm particle size) at a flow rate of 0.2 mL/min under a 12-min linear gradient. Mobile phase A consisted of 0.1% (*v*/*v*) formic acid (Beijing Inno Chem Science & Technology Co., Ltd., 270480010, Beijing, China) in water, and mobile phase B was methanol. The gradient program was as follows: 2% B (0–1.5 min), 2–85% B (1.5–4.5 min), 85–100% B (4.5–14.5 min), 100–2% B (14.5–14.6 min), and 2% B (14.6–16.6 min, equilibration). Mass spectrometry was performed using both the positive and negative ion modes with a spray voltage of 3.5 kV, a capillary temperature of 320 °C, a sheath gas flow rate of 35 psi, aux gas flow rate of 10 L/min, an S-lens RF level of 60, and an aux gas heater temperature of 350 °C.

### 2.4. Data Processing and Metabolite Identification

Raw UHPLC-MS/MS data were processed using XCMS (Xtracted Chromatograms and Mass Spectra) for metabolite peak detection, alignment, and quantification. Metabolite identification was performed by matching adduct ions with a mass tolerance of 10 ppm against the Human Metabolome Database (HMDB) spectral library. Background ions identified in blank samples were excluded prior to normalization of raw quantification values using the formula: Relative peak areas = Raw quantitative value of samples/(The sum of quantitative value of samples/The sum of quantitative value of QC1). Compounds exhibiting > 30% coefficient of variation (CV) in the QC sample relative to peak areas were filtered out, yielding final annotated metabolite identities and normalized abundances. All analyses were executed on a Linux platform (CentOS, version 6.6) using custom R (version 3.4.3)/Python pipelines (version 2.7.6).

### 2.5. Data Analysis

Statistical analyses were performed primarily in R language. Pearson correlation coefficients between differential metabolites were computed using cor () in R, with statistical significance (*p*-value ≤ 0.05) determined by cor.mtest() from the R stats package (version: 3.4.3). Partial least squares-discriminant analysis (PLS-DA) was conducted in the metaX software suite (version: 2.67) [35]. All data were tested for normality prior to statistical analysis using the Shapiro-Wilk test. Data satisfying normality assumptions (*p*-value > 0.05) were analyzed with Student’s *t*-test for comparisons between two groups. Univariate significance (*p*-value) was determined by Student’s *t*-test.

Metabolites were annotated using the HMDB (https://hmdb.ca/metabolites accessed on 5 June 2025), KEGG (https://www.genome.jp/kegg/pathway.html accessed on 5 June 2025) and LIPID MAPS (http://www.lipidmaps.org accessed on 5 June 2025) databases. The screening of differential metabolites primarily relied on three parameters: Variable Importance in the Projection (VIP), Fold Change (FC), and *p*-value. The VIP was derived from the first principal component of the PLS-DA model [36], indicating the contribution of a metabolite to group separation. The FC denotes the fold change, calculated as the ratio of the mean quantified values across all biological replicates in the comparison groups. The *p*-value, derived from the *T*-test [37], represents the significance level of the difference. Metabolites were identified as differential with thresholds set at VIP > 1.0, (FC > 1.5 or FC < 0.667), and *p*-value ≤ 0.05 [38,39,40].

Volcano plots visualizing −log_10_ (*p*-value) vs. log_2_ (FC) were generated using in R to identify metabolites of interest. Hierarchical clustering heatmaps were visualized using the pheatmap R package (version: 3.4.3). Significant correlations (*p*-value ≤ 0.05) were plotted with the corrplot package (version: 0.95). Functional annotation of metabolites and pathway analysis used the KEGG database. Metabolic pathway enrichment was assessed using the metric, Enrichment Ratio = (x/n)/(y/N), where x = number of significant metabolites in a pathway, *n* = total significant metabolites, y = number of metabolites annotated to the pathway in KEGG, and N = total annotated metabolites in the dataset. Pathways that satisfied *p*-value ≤ 0.05 after multiple testing correction were considered significantly enriched.

The Gene Set Enrichment Analysis (GSEA) was conducted on the KEGG entries based on quantitative changes in metabolites. The analysis calculates an Enrichment Score (ES), which quantifies the degree to which a metabolite set is concentrated at the extremes of the ranked list. Statistical significance is then evaluated through the *p*-value of the ES, the Normalized Enrichment Score (NES) (a size-normalized version of ES), and the False Discovery Rate (FDR), derived from NES. Pathways meeting the following criteria: |NES| > 1, *p*-value ≤ 0.05, and FDR < 0.25 were considered significantly enriched [41,42]. The analyses and data processing were primarily performed using Python (version: 3.5.0), while visualizations were generated with R language (version: 3.4.3). Detailed information on the software used is provided in Table S1.

## 3. Result

### 3.1. Data Quality Control and Metabolite Identification in Cauda Epididymal Fluid from Yak and Cattle

Distinct morphological differences were documented between yak and cattle epididymides. Our findings revealed that the length and weight of the yak epididymis are both lower than those of cattle at similar body weights (Figure S1A,B). In addition, the yak epididymis exhibited a vibrant red coloration due to dense vascular distribution, whereas the cattle epididymis appeared pale pink. Notably, both the caput and cauda of the yak epididymis demonstrated prominent enlargement, while their corpus (body) segment remained comparatively short and thick. Conversely, the cattle epididymis displayed moderate expansion at the head and tail portions with an elongated, slender corpus (Figure S1C,D).

In this study, the QC samples from yak and cattle epididymal cauda fluid exhibited a Pearson correlation coefficient of 0.994 (Figure S2A,B), confirming robust data integrity. The Partial Least Squares Discrimination Analysis (PLS-DA), a supervised discriminant analysis method, was validated through permutation testing to detect overfitting. Model reliability was defined by R^2^ (goodness-of-fit) exceeding Q^2^ (predictive ability), with the Q^2^ regression line intercepting the *Y*-axis below zero [43,44]. Our PLS-DA permutation results demonstrated R^2^ > Q^2^ and negative *Y*-axis intercepts for Q^2^ regression lines under both positive and negative ion modes (Figure S2C,D), validating model reliability for subsequent analyses.

**Figure 1:**
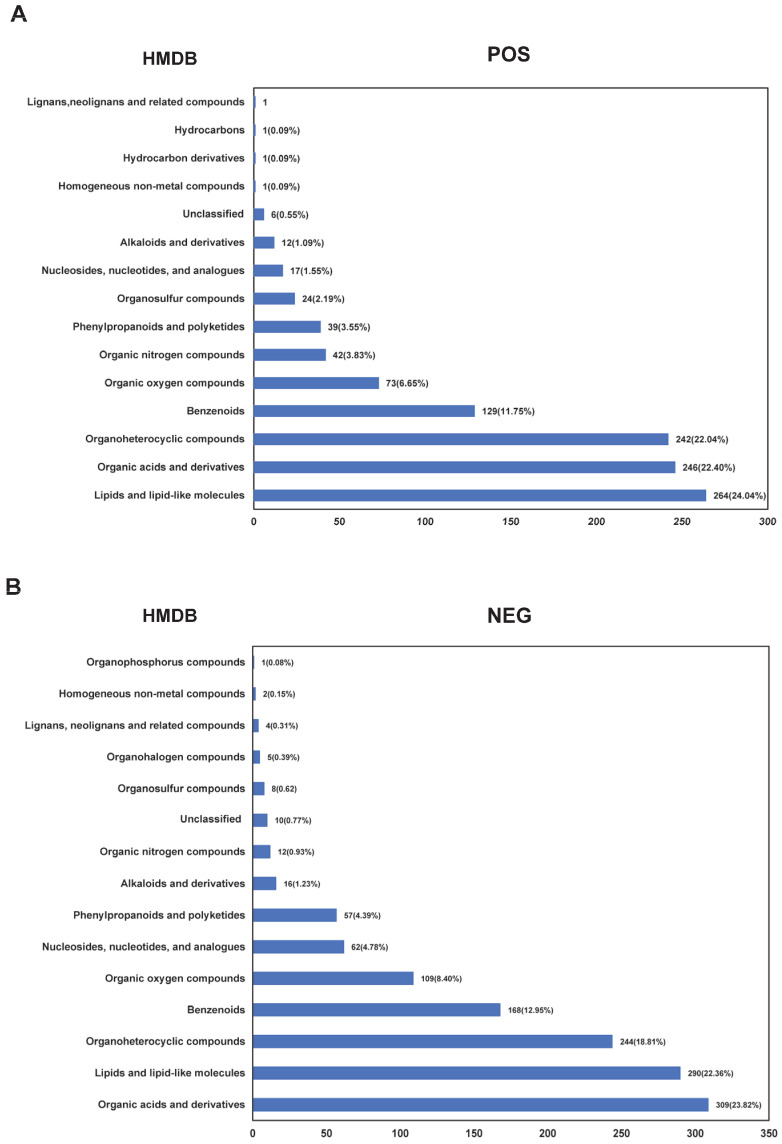
Statistical analysis of metabolites in epididymal tail fluid of yak and cattle based on the HMDB database. The HMDB classification and annotation statistics for metabolites under positive (**A**) and negative (**B**) ion modes.

A total of 1098 kinds of metabolites were identified in the cauda epididymal fluid from yak and cattle in positive ion mode, annotated by the HMDB database, spanning fifteen major categories. Among these, lipids and lipid-like molecules (264 types of metabolites, 24.04%), organic acids and derivatives (*n* = 246, 22.40%), and organoheterocyclic compounds (*n* = 242, 22.04%), collectively accounted for the most significant proportions. Other categories included benzenoids (*n* = 129, 11.75%), organic oxygen compounds (*n* = 73, 6.65%), organic nitrogen compounds (*n* = 42, 3.83%), phenylpropanoids and polyketides (*n* = 39, 3.55%), organosulfur compounds (*n* = 24, 2.19%), nucleosides, nucleotides, and analogues (*n* = 17, 1.55%), alkaloids and derivatives (*n* = 12, 1.09%), hydrocarbons (*n* = 1, 0.09%), lignans, neolignans and related compounds (*n* = 1, 0.09%), hydrocarbon derivatives (*n* = 1, 0.09%), and homogeneous non-metal compounds (*n* = 1, 0.09%), with an additional six metabolites (0.55%) classified as unclassified compounds (Figure 1A; Tables S2 and S3). In contrast, a total of 1297 metabolites were identified in the cauda epididymal fluid of yak and cattle in the negative ion mode and annotated by the HMDB database, classified into 17 major categories. These metabolites were categorized as follows: organic acids and derivatives (*n* = 309, 23.82%), lipids and lipid-like molecules (*n* = 290, 22.26%), organoheterocyclic compounds (*n* = 244, 18.81%), benzenoids (*n* = 168, 12.95%), organic oxygen compounds (*n* = 109, 8.40%), nucleosides, nucleotides, and analogues (*n* = 62, 4.78%), phenylpropanoids and polyketides (*n* = 57, 4.39%), alkaloids and derivatives (*n* = 16, 1.23%), organic nitrogen compounds (*n* = 12, 0.93%), organosulfur compounds (*n* = 8, 0.62%), organohalogen compounds (*n* = 5, 0.39%), lignans, neolignans and related compounds (*n* = 4, 0.31%), homogeneous non-metal compounds (*n* = 2, 0.15%), organophosphorus compounds (*n* = 1, 0.08%), and unclassified compounds (*n* = 10, 0.77%) (Figure 1B; Tables S4 and S5).

**Figure 2:**
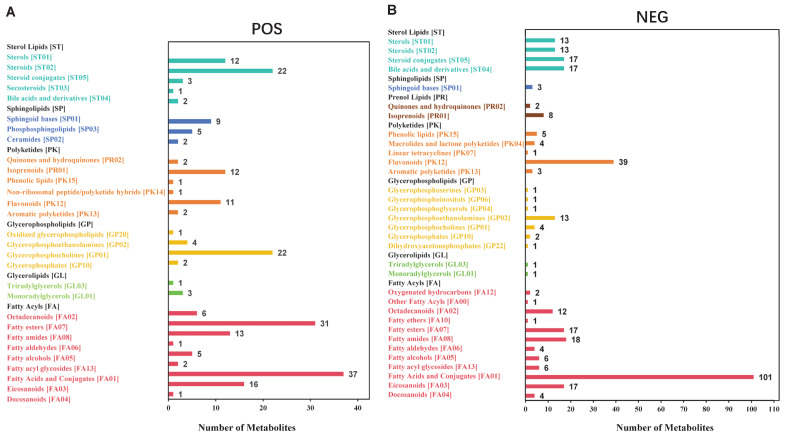
Statistical results of lipid metabolites in the cauda epididymis of yaks and cattle. Classification of lipid metabolites under positive (**A**) and negative (**B**) ion modes in cauda epididymis of yaks and cattle according to the LIPID MAPS database. The *x*-axis represents the number of metabolites. The *y*-axis represents the annotated LIPID MAPS lipid classification. This figure shows the number of metabolites corresponding to the main class level under the eight major lipid categories in the LIPID MAPS.

The LIPID MAPS is a comprehensive database containing biologically relevant lipid structures and annotations [45,46]. Based on the LIPID MAPS classification and annotation of lipid metabolites in the cauda epididymal fluid of both yak and cattle, the results indicated the presence of six major lipid categories in positive ion mode, including fatty acyls, glycerolipids, glycerophospholipids, sphingolipids, sterol lipids, and polyketides. Within these, the category containing the most subclasses was fatty acyls (*n* = 112), and the subclass with the highest number of metabolites was fatty acids and conjugates (*n* = 37) (Figure 2A; Tables S6 and S7). In contrast, the lipids in the negative ion mode included seven major categories: fatty acids, glycerolipids, glycerophospholipids, sphingolipids, sterol lipids, polyketides, and prenol lipids. Among these, fatty acyls contained the most subclasses (*n* = 189), and the subclass with the highest number of metabolites was fatty acids and conjugates (*n* = 101) (Figure 2B; Tables S8 and S9).

### 3.2. Identification of Differential Metabolites in Cauda Epididymal Fluid Between Yak and Cattle

**Figure 3:**
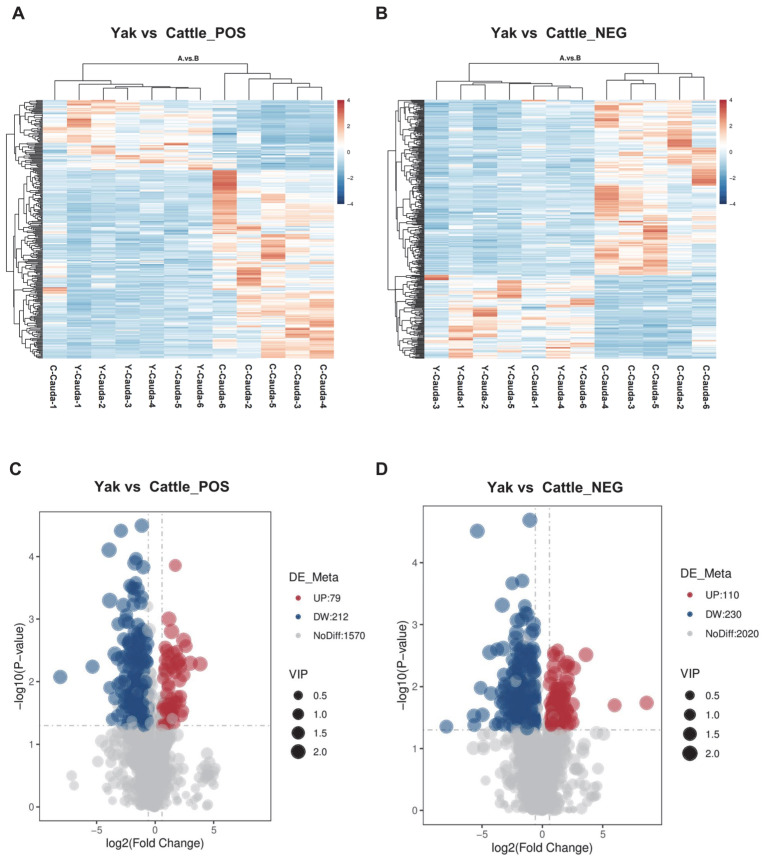
Analysis of differential metabolites in cauda epididymal fluid from yak and cattle. Clustering heatmap of differentially expressed metabolites in positive (**A**) and negative (**B**) ion modes. Y-Cauda-1 to Y-Cauda-6 represent six biologically independent samples of epididymal cauda fluid from yak and C-Cauda-1 to C-Cauda-6 represent six biologically independent samples of epididymal cauda fluid from cattle. The metabolites were clustered along the vertical axis, while sample groups were arranged along the horizontal axis. Shorter dendrogram branches indicate higher similarity between the connected elements (metabolites or samples). The volcano plots comparing significantly differentially expressed metabolites in positive (**C**) and negative (**D**) ion modes: The *x*-axis represents log_2_ (fold change) (log_2_FC) in metabolite expression between two groups, and the *y*-axis indicates the level of differential significance (−log_10_ (*p*-value)). Each point denotes an individual metabolite, with point size corresponding to VIP values. The metabolites showing statistically significant upregulation were highlighted in red, and those with significant downregulation were depicted in blue.

The clustering heatmap of total differential metabolites across 12 samples is presented in Figure 3A,B. Among the 1861 metabolites identified in positive ion mode, using the screening criteria of VIP > 1.0, FC > 1.5 or FC < 0.667, and *p*-value ≤ 0.05 for significant differences, 79 metabolites were significantly upregulated, while 212 were significantly downregulated in yak cauda epididymal fluid compared with cattle (Figure 3C; Table S10). In addition, among the 2360 metabolites identified in the negative ion mode, 110 were significantly upregulated and 230 were significantly downregulated in yak cauda epididymal fluid compared with cattle (Figure 3D; Table S11).

**Figure 4:**
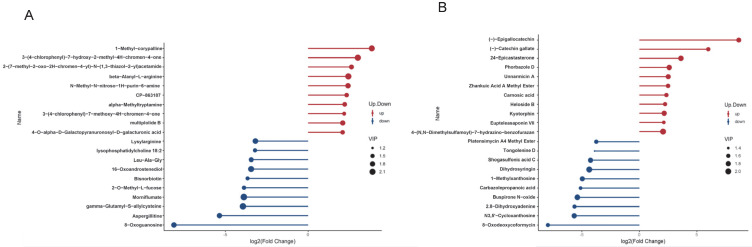
Lollipop plots for significantly differentially expressed metabolites in positive (**A**) and negative (**B**) ion modes. The lollipop plots visualize the top ten up/downregulated metabolites based on absolute fold-change magnitude. The *x*-axis represents log_2_FC values and the *y*-axis lists metabolite names. The point size corresponds to VIP values, with the red points denoting significantly upregulated metabolites and the blue points indicating significantly downregulated metabolites.

Lollipop plots were generated to visualize the 12 metabolites exhibiting the most significant fold changes in positive and negative ion modes (Figure 4A,B). In the comparison of yak vs. cattle cauda epididymal fluid in positive ion mode, the three metabolites with the most significant upregulation were 1-Methyl-corypalline, 3-(4-chlorophenyl)-7-hydroxy-2-methyl-4H-chromen-4-one, and 2-(7-methyl-2-oxo-2H-chromen-4-yl)-N-(1,3-thiazol-2-yl) acetamide. Conversely, the three metabolites with the most significant downregulation were 8-Oxoguanosine, Aspergillitine, and Gamma-glutamyl-s-allylcysteine (Figure 4A). In the negative ion mode, for the yak vs. cattle cauda epididymal fluid comparison, the three metabolites with the most significant upregulation were (-)-Epigallocatechin, Catechin gallate, and 24-Epicastasterone. The three metabolites with the most significant downregulation were 8-Oxodeoxycoformycin, N3,5′-Cycloxanthosine, and 2,8-Dihydroxyadenine (Figure 4B).

To assess the coordinated or antagonistic relationships between different metabolites, Pearson correlation coefficients were calculated among all significant differential metabolites in the cauda epididymal fluid of yak vs. cattle to evaluate the co-variation trends between metabolites. The top 20 significant differential metabolites ranked by ascending *p*-value, in positive and negative ion mode, are shown (Figure S3A,B; Tables S12 and S13). A correlation value of 1 indicates a perfect positive correlation (red), while −1 indicates a perfect negative correlation (blue). Uncolored areas represent correlations with *p*-values > 0.05. As shown in the figure, the vast majority of significant differentially expressed metabolites exhibited positive correlations. These results are also visualized in the chord diagrams (Figure S3C,D).

### 3.3. Functional Enrichment Analyses of Differential Metabolites in Cauda Epididymal Fluid Between Yak and Cattle

**Figure 5:**
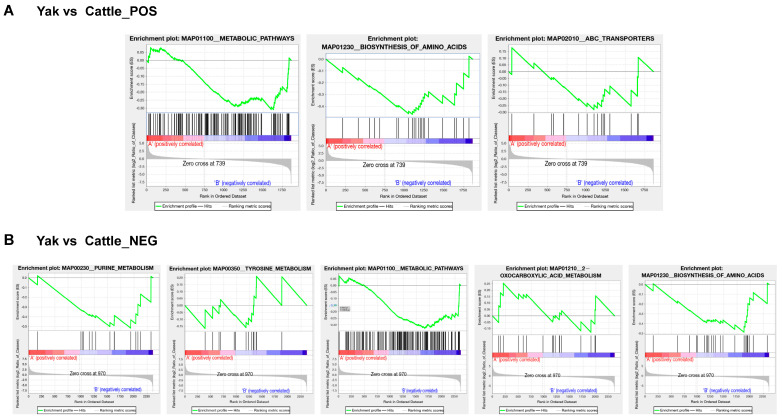
The Gene Set Enrichment Analysis (GSEA) of significantly altered cationic (**A**) and anionic metabolites (**B**). The upper section displays an enrichment score (ES) line graph for functional terms. The peak in this graph represents the ES value of a metabolite functional set. A higher absolute ES value indicates stronger enrichment, where a positive ES signifies enrichment of the functional set-in upregulated metabolites, while a negative ES denotes enrichment in downregulated metabolites. In the middle section, the positions of functional set members within the ranked expression list are visualized: the black vertical marks indicate members of the functional set; and the colored bars represent the ranked expression list (ordered by correlation), with red for upregulated members and blue for downregulated ones. The lower section shows the log_2_FC values of all members in the expression set.

To investigate the functional implications of significant metabolic differences, the KEGG pathway enrichment analysis was conducted via the GSEA. By applying the GSEA to the KEGG entries based on quantitative metabolite changes, significantly altered metabolites in positive ion mode were enriched in three pathways: ‘Biosynthesis of amino acids’ (map01230), ‘Metabolic pathways’ (map01100), and ‘ABC transporters’ (map02010). Significantly altered metabolites in the negative ion mode were enriched in five pathways, including ‘2-Oxocarboxylic acid metabolism’ (map01210), ‘Tyrosine metabolism’ (map00350), Biosynthesis of amino acids (map01230), Purine metabolism (map00230), and ‘Metabolic pathways’ (map01100) (Figure 5A,B; Tables S14–S19).

### 3.4. Identification of Differential Metabolites Related to Sperm Quality, Function or Metabolism

**Figure 6:**
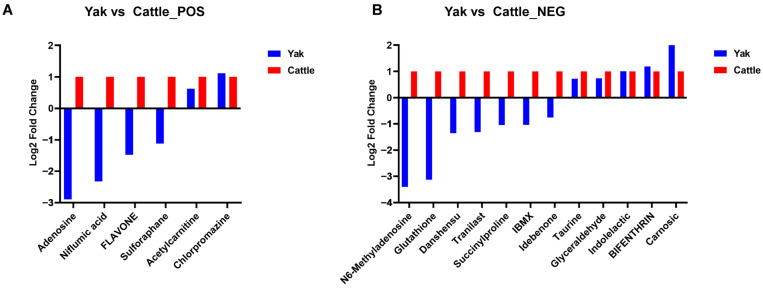
The fold-change of significantly differential expressed metabolites related to sperm mobility, mature or function in epididymal fluid between yak and cattle in positive (**A**) and negative (**B**) ion modes, the *x*-axis indicates the names of metabolites and the *y*-axis represents log_2_FC values.

Among the differential metabolites between yak and cattle epididymal cauda fluid, six metabolites in positive ion mode and twelve metabolites in negative ion mode were associated with sperm quality, mature function or metabolism (Figure 6; Table 1 and Table 2). Among these, five metabolites (Flavone, Sulforaphane, Glutathione, Danshensu, and Idebenone) were reported to enhance sperm motility through their antioxidant capacity; one metabolite, Indolelactic acid, was found to reduce sperm count by elevating oxidative stress levels; and five metabolites (Adenosine, Niflumic acid, Chlorpromazine, BIFENTHRIN, and IBMX) were linked to sperm acrosome reaction, capacitation, or chemotaxis. In addition, three metabolites (Acetylcarnitine, Taurine, and Carnosic acid) were documented to improve sperm energy metabolism; one metabolite (N6-Methyladenosine) was associated with sperm DNA methylation; and another metabolite (Glyceraldehyde 3-phosphate) was implicated in sperm flagellar movement. Additionally, two metabolites (Tranilast, Succinylproline) were clinically reported to affect sperm motility, though their mechanisms remain unclear.

## 4. Discussion

Previous research indicates that the epididymal lumen maintains an acidic microenvironment, which is crucial for maintaining spermatozoa in a quiescent state and preventing premature capacitation [75,76]. In this study, under the positive ion mode, ‘organic acids and derivatives’ constituted the largest category of metabolites identified in yak epididymal fluid. This finding suggests that such substances likely play a crucial role in maintaining the low pH environment within the yak epididymal cauda fluid.

Lipid-like molecules also constitute a significant portion of the metabolome in the epididymal cauda fluid of both yaks and cattle. Under positive ion mode, 411 lipid-like metabolite features were identified, while 602 were found under negative ion mode. Database annotation revealed that fatty acids constituted the most abundant subclass at the second-level classification in both modes. This suggests that fatty acids may play a crucial role in sperm maturation within the yak epididymis. Existing research indicates that fatty acids are vital for sperm maturation in humans, pigs, and cattle. For example, unsaturated fatty acids enhance boar sperm motility after storage at 6 °C [77]. Short-chain fatty acids have been established to modulate human sperm migration via olfactory receptor 51E2 activity [78]. Furthermore, saturated fatty acids accelerate the linear motility of bull sperm through mitochondrial ATP production [79]. We propose that the altered fatty acid composition observed in yak epididymal fluid, particularly the relative deficit in certain polyunsaturated fatty acids, may contribute to differences in membrane properties and metabolic microenvironment compared with cattle. These differences warrant further investigation to determine their potential implications for sperm function adaptation in high-altitude environments.

During storage in the cauda epididymis, sperm maintain a chronic hypoxic state to minimize metabolic activity and prevent premature capacitation [17,80]. The cauda epididymal environment, rich in specific proteins and enzymes and with regulated pH, contributes to maintaining this quiescence. Comparative metabolomic profiling between yaks and cattle revealed differential abundance of certain metabolites in the cauda epididymal fluid. Five antioxidant metabolites, including Glutathione, Flavone, Sulforaphane, Danshensu, and Idebenone [50,51,52,53,54,58,59,60,61,66,68,81], exhibited significantly decreased levels in yak cauda epididymal fluid compared to that of cattle. Conversely, a pro-oxidant metabolite, Indolelactic acid [71], showed a marked increase in yak cauda epididymal fluid. While oxidative stress is known to damage sperm membrane structure, induce lipid peroxidation, impair energy metabolism, and accelerate DNA fragmentation and apoptosis [82,83], the functional implications of these observed metabolic differences between species require further targeted investigation. Future studies aimed at validating these findings could explore the targeted supplementation of these antioxidants or inhibitors of the identified pro-oxidants into yak frozen or fresh semen to assess their potential in enhancing sperm vitality and quality by improving the antioxidant and oxidant balance.

Under physiological conditions, sperm capacitation primarily occurs within the endometrium and oviduct; however, premature capacitation occurs when this process initiates in suboptimal microenvironments or concludes prematurely under poorly regulated in vitro conditions [82,84]. During natural fertilization, the interaction between sperm and the zona pellucida of the oocyte triggers the acrosome reaction, releasing acrosomal enzymes to dissolve the zona and enable sperm-egg binding. The spontaneous release of acrosomal enzymes prior to zona pellucida contact or other physiological inducers is classified as a spontaneous acrosome reaction [84,85]. Both processes cause sperm to prematurely lose their egg-binding capacity, compromising fertilization success. Our comparative metabolomic analysis revealed differential abundance of several metabolites in yak cauda epididymal fluid compared with cattle. Four metabolites with significantly different abundances in yak cauda epididymal fluid compared with cattle, including Adenosine, Niflumic acid, Chlorpromazine, and Bifenthrin, which have been reported in existing literature to influence premature capacitation and spontaneous acrosome reaction [47,48,49,56,72,73], showed significant abundance differences. Among these metabolites, Adenosine and Niflumic acid were significantly downregulated in yak cauda epididymal fluid, while Chlorpromazine and Bifenthrin were significantly upregulated. Additionally, one metabolite, IBMX, previously associated with promotion of premature capacitation [65], was significantly downregulated in yak cauda epididymal fluid. This observed disparity in the abundance of metabolites with purported opposing functions suggests a compositional shift in the yak epididymal microenvironment. The metabolic differences identified here provide a basis for future targeted research into their functional significance regarding sperm capacitation regulation in yaks. Further investigation via in vitro fertilization (IVF) assays is necessary to determine whether these metabolic changes directly compromise yak sperm fertilizing capacity.

Sperm remain in a quiescent state within the epididymis for extended periods. During this period, mitochondria minimize the ATP synthesis rates and inhibit oxidative phosphorylation activity to prevent premature energy depletion [17,80]. Our comparative metabolomic analysis revealed that the levels of three metabolites previously associated with mitochondrial energy metabolism in other systems, including Taurine, Carnosic acid, and Acetylcarnitine [26,55,69,74], were significantly upregulated in yak cauda epididymal fluid compared to that of cattle. This finding points to a species-specific difference in the metabolic composition of the epididymal microenvironment between yaks and cattle. The observed metabolic differences highlight a comparative divergence between yaks and cattle and provide a basis for future targeted research into their functional significance regarding sperm energy regulation.

N6-Methyladenosine (m6A), the most prevalent post-transcriptional modification in eukaryotic mRNA and non-coding RNA (ncRNA), regulates the RNA fate to influence gene expression and has been associated with sperm motility and energy metabolism [57]. Studies have reported differential m6A levels across the caput, corpus, and cauda epididymis of yaks, with significantly lower levels observed in the cauda compared with the corpus [86]. In the present study, we found that m6A levels in yak cauda epididymal fluid were significantly lower than those in cattle, suggesting a species-specific difference in epididymal m6A modification. This comparative metabolomic profiling highlights a distinct m6A landscape between yak and cattle, which may serve as a foundation for further targeted research on its regulatory role in sperm maturation. However, direct functional inferences regarding sperm function and fertility cannot be drawn from these untargeted findings and require subsequent validation, especially given the species-specific nature of processes such as sperm migration and fertilization in ruminants.

The Gene Set Enrichment Analysis (GSEA) effectively addresses the limitations of traditional enrichment methods in information mining, providing a more comprehensive interpretation of the regulatory roles of functional units. Its core principle involves using predefined metabolite sets to rank metabolites based on their differential expression magnitude between two sample groups [41,42]. The GSEA analysis indicated that the biosynthesis of amino acids was one of the pathways significantly enriched with differential metabolites between yak and cattle epididymal cauda fluid. Amino acid biosynthesis represents one of the core biological processes in living organisms. Beyond their fundamental role in protein synthesis, amino acids function as signaling molecules and are indispensable for diverse metabolic functions including gluconeogenesis, lipogenesis, ATP generation, and nucleotide base synthesis. Additionally, amino acids play significant roles in cellular protection against oxidative stress [87,88]. Studies have indicated that the biosynthetic capacity of certain amino acids is significantly reduced in human azoospermic patients [89]. Our research further demonstrated that, compared with cattle epididymal cauda fluid, the levels of five amino acids in yak epididymal cauda fluid–L-Saccharopine, L-Glutamine, S-Adenosylhomocysteine, L-Arogenate, and L-Valine-were significantly decreased. These findings highlight distinct metabolomic profiles between yaks and cattle, which may reflect species-specific metabolic adaptations or regulatory mechanisms. Further investigation is warranted to determine the precise roles of these specific amino acids in yak sperm metabolism and whether the observed differences are associated with specific physiological traits in yaks.

It should be noted that although epididymal fluid plays a crucial role in sperm maturation, spermatozoa are subsequently resuspended in seminal plasma and enter the female reproductive tract. Therefore, the metabolites identified in this study as potential regulators of yak sperm quality and function in the epididymis may be modified, diluted, or even eliminated during ejaculation and entry into the female reproductive tract. Future research should include further quantification of these metabolites in seminal plasma, as well as more rigorous in vitro and in vivo experiments to verify whether these metabolites indeed influence yak sperm quality or function. Additionally, it is important to emphasize that limitations such as sample size, variations in feeding management levels, and fluctuations in environmental conditions may have affected the experimental results. Subsequent studies should pay particular attention to these factors.

## 5. Conclusions

This study analyzed and compared the metabolomes of the epididymal cauda in yak and cattle. The findings provide foundational data and novel research perspectives for in-depth elucidation of the molecular mechanisms of sperm maturation in yaks, with potential implications for improving sperm motility and optimizing assisted reproductive technologies, such as cryopreservation, in vitro fertilization, and artificial insemination. However, given the species-specific nature of metabolic influences on sperm function, direct extrapolations regarding fertility outcomes should be avoided until further validation. Future research should prioritize in vitro validation of the specific regulatory roles of these metabolites in yak sperm functionality and explore their practical applications in assisted reproduction, thus contributing to the development of targeted strategies for enhancing yak reproductive efficiency.

## Figures and Tables

**Table 1:** Differential metabolites related to sperm quality, function or metabolism in epididymal fluid between yak and cattle detected in positive ion mode.

Name	Formula	Classification	Log_2_FC	*p*-Value	Function Related to Sperm
Adenosine	C_10_H_13_N_5_O_4_	Nucleosides, nucleotides, and analogues	−2.8923	0.0145	Inhibition of spontaneous acrosome reaction [47]
Niflumic acid	C_13_H_9_F_3_N_2_O_2_	Benzenoids	−2.3234	0.0470	Blocking sperm acrosome reaction and chemotaxis [48,49]
FLAVONE	C_15_H_10_O_2_	Phenylpropanoids and polyketides	−1.4766	0.0042	Protecting against acrosome damage, capacitation, apoptosis, and prevents lipid peroxidation during sperm cryopreservation [50]
Sulforaphane	C_6_H_11_NOS_2_	Organosulfur compounds	−1.1168	0.0246	Reducing oxidative stress and endoplasmic reticulum stress in sperm, thus improving sperm motility and fertilization rates in frozen semen [51,52,53,54]
Acetylcarnitine	C_9_H_17_NO_4_	Lipids and lipid-like molecules	0.6234	0.0375	Enhancing sperm energy metabolism and motility while reducing apoptosis [26,55].
Chlorpromazine	C_17_H_19_ClN_2_S	Organoheterocyclic compounds	1.1107	0.0110	Inhibition of epididymal sperm capacitation and acrosomal reaction [56]

**Table 2:** Differential metabolites related to sperm quality, function or metabolism in epididymal fluid between yak and cattle detected in the negative ion mode.

Name	Formula	Classification	Log_2_FC	*p*-Value	Function Related to Sperm
N6-Methyladenosine	C_11_H_15_N_5_O_4_	Nucleosides, nucleotides, and analogues	−3.3979	0.0177	Increased N6-Methyladeno-sine content is a risk factor for asthenozoospermia and affects sperm motility [57].
Glutathione	C_10_H_17_N_3_O_6_S	Organic acids and derivatives	−3.1306	0.0152	Increasing sperm count, motility and fertilization rate by reducing ROS levels [58,59,60].
Danshensu	C_9_H_10_O_5_	Phenylpropanoids and polyketides	−1.3530	0.0481	Improving seminal quality and antioxidant capacity [61].
Tranilast	C_18_H_17_NO_5_	Phenylpropanoids and polyketides	−1.3146	0.0024	Increasing sperm count [62,63].
Succinylproline	C_9_H_13_NO_5_	Organic acids and derivatives	−1.0481	0.0248	Inhibiting spermatozoa motility [64].
IBMX	C_10_H_14_N_4_O_2_	Organoheterocyclic compounds	−1.0398	0.0028	Ameliorate sperm capacitation and motility and mobility [65].
Idebenone	C_19_H_30_O_5_	Lipids and lipid-like molecules	−0.7619	0.0246	Improving post thaw sperm quality by mitigating oxidative stress [66,67,68].
Taurine	C_2_H_7_NO_3_S	Organic acids and derivatives	0.7165	0.0287	Enhancing sperm mitochondrial energy metabolism [69].
Glyceraldehyde 3-phosphate	C_3_H_7_O_6_P	Organic oxygen compounds	0.7332	0.0473	Facilitating sperm flagellum motility [70].
Indolelactic acid	C_11_H_11_NO_3_	Organoheterocyclic compounds	1.0075	0.0377	Increasing ROS level, decreasing antioxidant activity and reducing sperm number [71].
BIFENTHRIN	C_23_H_22_ClF_3_O_2_	Benzenoids	1.1856	0.0299	Diminishing motility, spontaneous acrosome reaction, and capacitation in sperm [72,73].
Carnosic acid	C_20_H_28_O_4_	Lipids and lipid-like molecules	2.3463	0.0296	Improving the quality and mitochondrial function of frozen-thawed sperm [74].

## Data Availability

The raw data of metabolomic sequencing were deposited in the MetaboLights database (https://www.ncbi.nlm.nih.gov/bioproject, accessed on 20 July 2025) with the accession number MTBLS13067. Other data presented in this study are included in the published article and Supplementary Materials. Further inquiries can be directed to the corresponding author.

## Supplementary Materials

The following supporting information can be downloaded at: https://www.mdpi.com/article/10.3390/ani15192861/s1, Figure S1. Morphology analysis of yak and cattle epididymis. (A,B) The lengths (C) and weights (D) of epididymis from yak and cattle. A total of six yak epididymides and six cattle epididymides were used for data analysis, with each epididymis originating from an independent individual. * *p*-value < 0.05 and *** *p*-value < 0.001, Student’s *t*-test. (C,D) Representative images of yak (C) and cattle (D) epididymis. The caput, corpus, and cauda of the epididymis are labeled in the figure. The two epididymides (upper and lower) in each panel originate from the same individual (*n* = 6). Figure S2. The result of data quality control. (A,B) Quality controlled (QC) correlation analysis of epididymal cauda fluid from yaks and cattle in positive (A) and negative (B) ion modes. The x and y axes represent the QC samples of corresponding species, with numbers indicating the Pearson correlation coefficients. (C,D) Validation plots of the Partial Least Squares Discrimination Analysis (PLS-DA) in positive (C) and negative (D) ion modes. The *x*-axis denotes the correlation between randomly permuted group Y and original group Y while the *y*-axis represents scores for R^2^ (goodness-of-fit) and Q^2^ (predictive ability). Figure S3. Correlation analysis of differential metabolites. (A,B) Correlation heatmap of differential metabolites in positive (A) and negative (B) ion modes. This visualization displays the top 20 differentially expressed metabolites, selected by ranking pairwise Pearson correlation coefficients between all differential metabolites in ascending order of *p*-value significance. Correlations range from +1 (indicating a perfect positive correlation, shown in red) to −1 (indicating a perfect negative correlation, shown in blue). Areas with no color shading represent non-significant correlations (*p*-value > 0.05). (C,D) Chord diagram of differentially expressed metabolites in positive (C) and negative (D) ion modes. The top 20 differentially expressed metabolites, ranked by ascending *p*-value, were selected based on pairwise Pearson correlation coefficients for visualization in this chord diagram. Each node represents a metabolite. The node size corresponds to the magnitude of the log-transformed fold change (FC) value for that metabolite. The node color indicates the metabolite’s class. The color of the curved lines connecting nodes represents the correlation between metabolites: blue indicates a negative correlation, while red indicates a positive correlation. Table S1. The information of software used in this study. Table S2. List of the HMDB-annotated metabolites detected in the positive ion mode. Table S3. Classification statistics of the HMDB for metabolites detected in the positive ion mode. Table S4. List of the HMDB-annotated metabolites detected in the negative ion mode. Table S5. Classification statistics of the HMDB for metabolites detected in the negative ion mode. Table S6. List of the LIPID MAPS annotation results for metabolites detected in the positive ion mode. Table S7. Classification statistics of LIPID MAPS for metabolites detected in the positive ion mode. Table S8 List of LIPID MAPS annotation results for metabolites detected in the negative ion mode. Table S9 Classification statistics list of the LIPID MAPS for metabolites detected in the negative ion mode. Table S10. List of significant differential metabolites analysis in the positive ion mode. Y Cauda1 to Y Cauda6 represent six biologically independent samples of epididymal cauda fluid from yak and C Cauda1 to C Cauda6 represent six biologically independent samples of epididymal cauda fluid from cattle. The same as below. Table S11. List of significant differential metabolites detected in the negative ion mode. Table S12. List of significant differential metabolites detected in the positive ion mode. Table S13. List of significant differential metabolites detected in the negative ion mode. Table S14. The KEGG classification list of differential metabolites detected in the positive ion mode. Table S15. The KEGG classification list of differential metabolites detected in the negative ion mode. Table S16. The KEGG enrichment list of differential metabolites detected in the positive ion mode. Table S17. The KEGG enrichment list of differential metabolites detected in the negative ion mode. Table S18. The GSEA enrichment results detected in the positive ion mode. Table S19. The GSEA enrichment results detected in the negative ion mode.

## Abbreviations

The following abbreviations are used in this manuscript:

HMBD	Human Metabolome Database
KEGG	Kyoto Encyclopedia of Genes and Genomes
PBS	Phosphate Buffered Saline
QC	Quality Control
CV	Coefficient of variation
PLS-DA	Partial least squares-discriminant analysis
VIP	Variable Importance in the Projection
FC	Fold Change
GSEA	Gene Set Enrichment Analysis
ES	Enrichment Score
NES	Normalized Enrichment Score
FDR	False Discovery Rate
PLS-DA	Partial Least Squares Discrimination Analysis
R^2^	Goodness-of-fit
Q^2^	Predictive ability
ncRNA	non-coding RNA
XCMS	Xtracted Chromatograms and Mass Spectra
