# Supplementary figures and images for: Metabolomic Analysis of Cauda Epididymal Fluid in Yaks and Cattle
Source: Animals (Basel). 2025 Sep 30;15(19):2861. doi: 10.3390/ani15192861 (PMC12524237; doi:10.3390/ani15192861)

**A**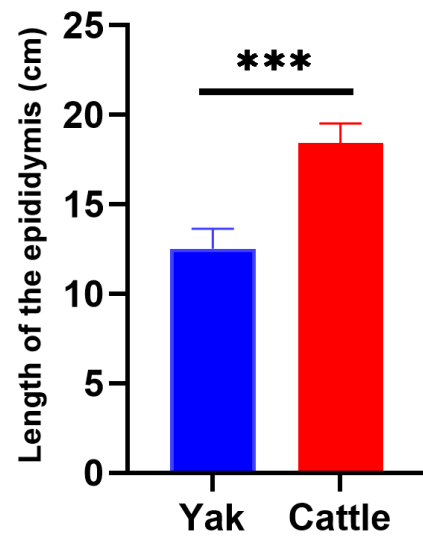**B**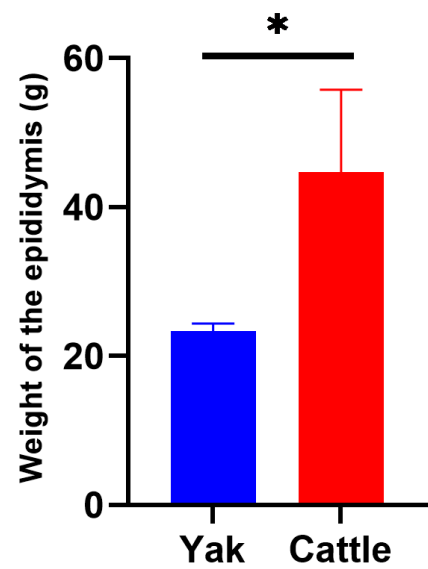**C**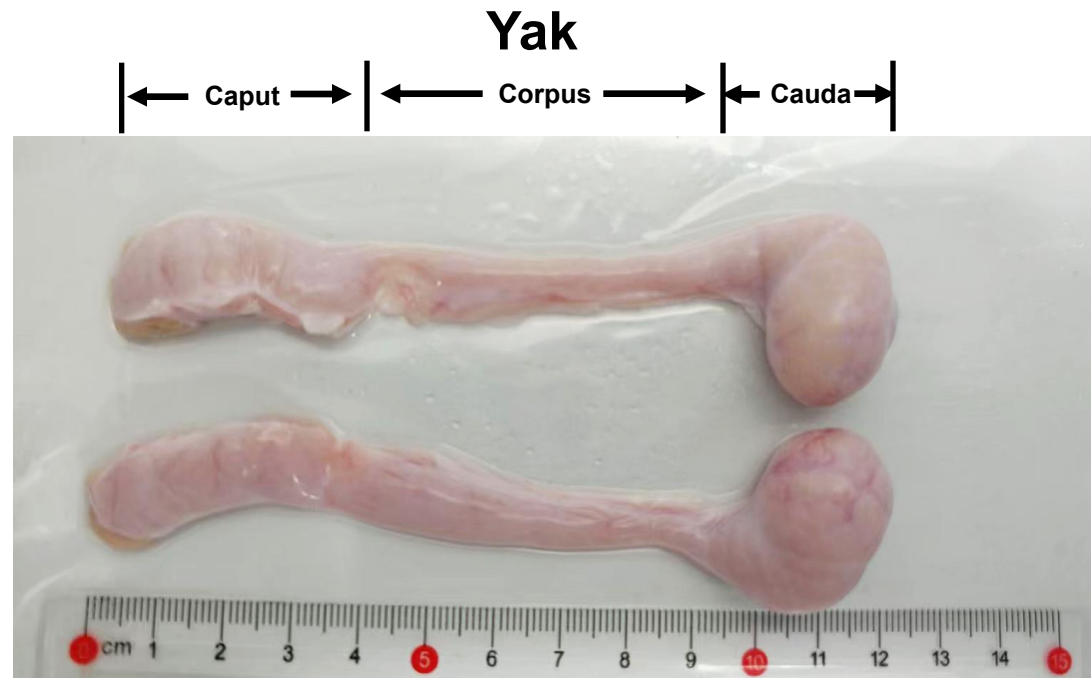**D**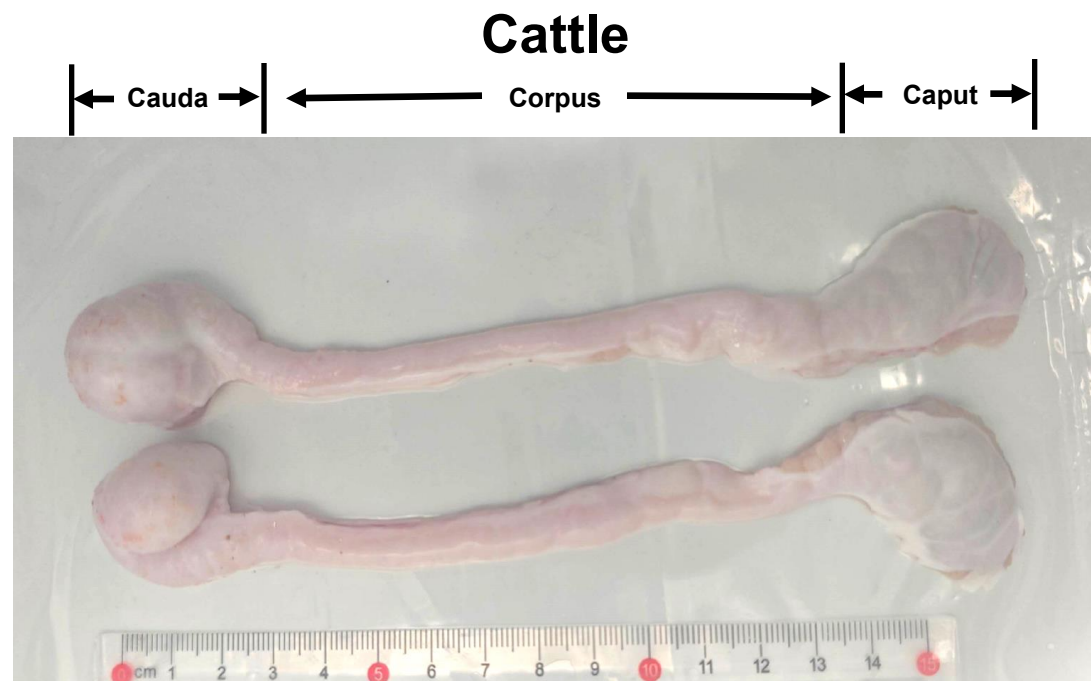

Supplement: Supplementary file 1 [file animals-15-02861-s001.zip › Figure S1.pdf]

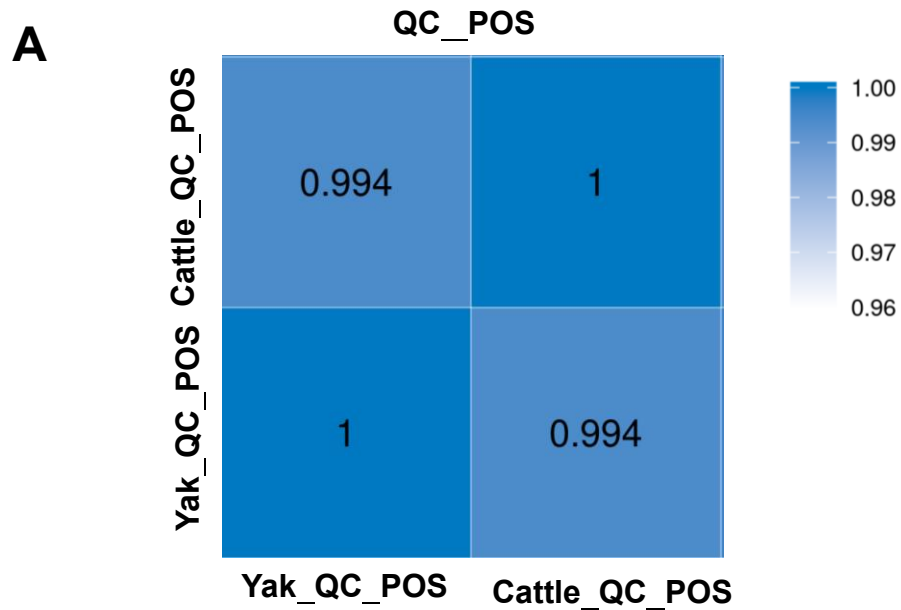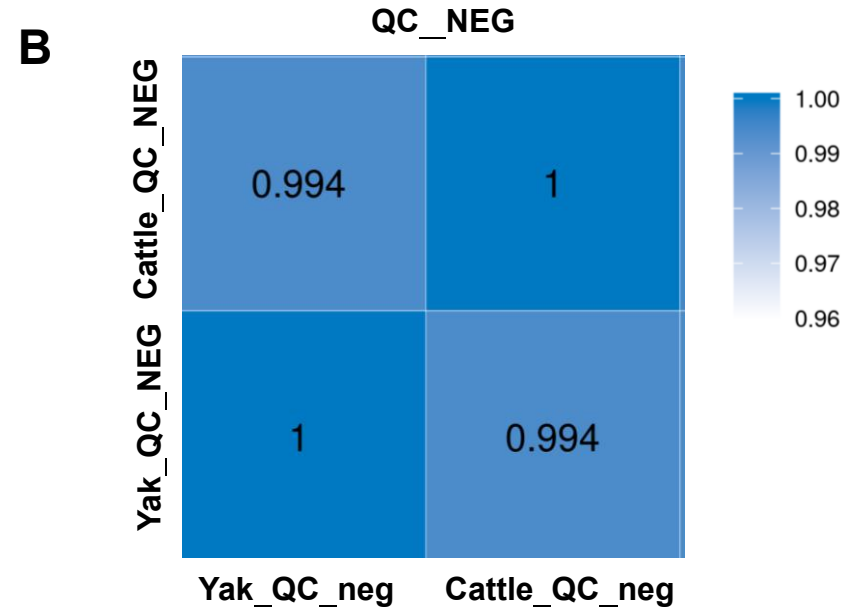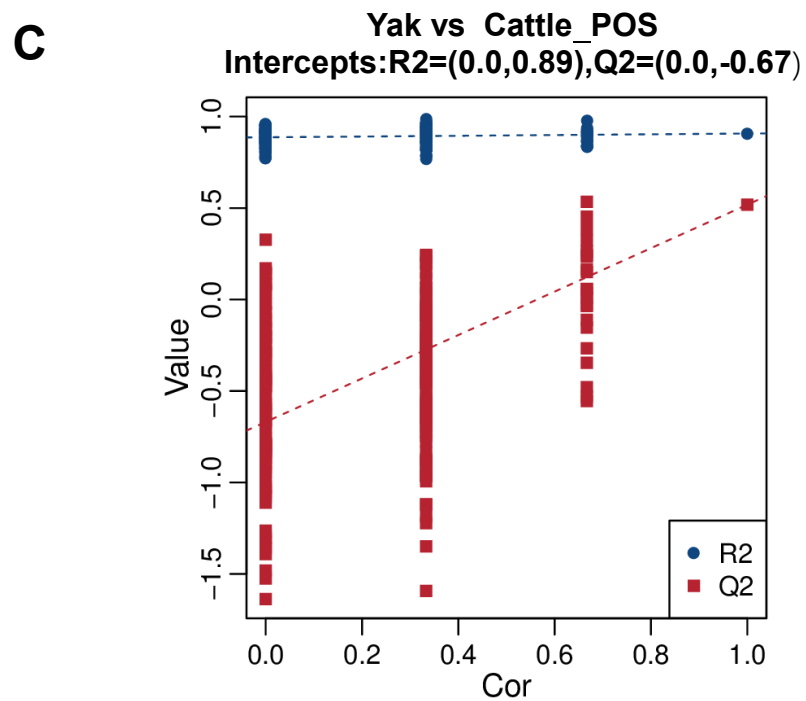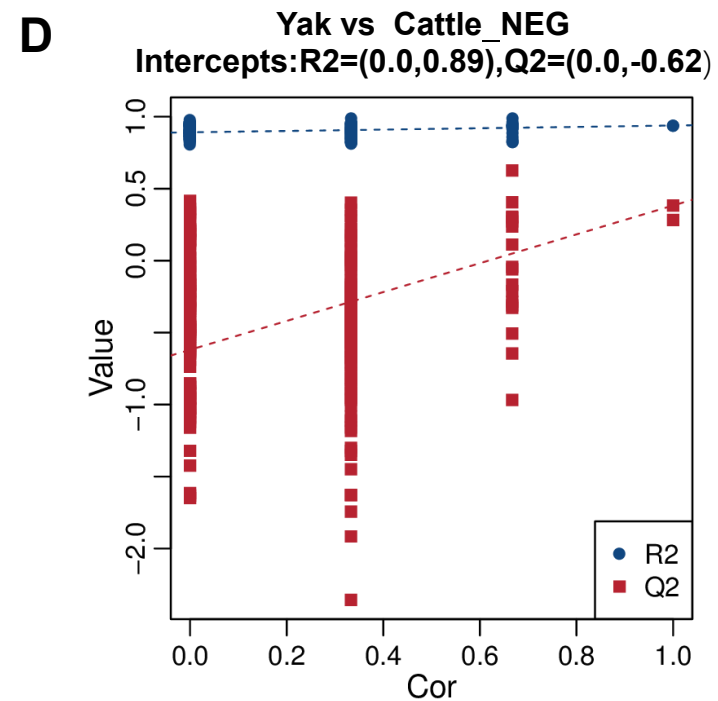

Supplement: Supplementary file 1 [file animals-15-02861-s001.zip › Figure S2.pdf]

A

Yak vs Cattle\_POS

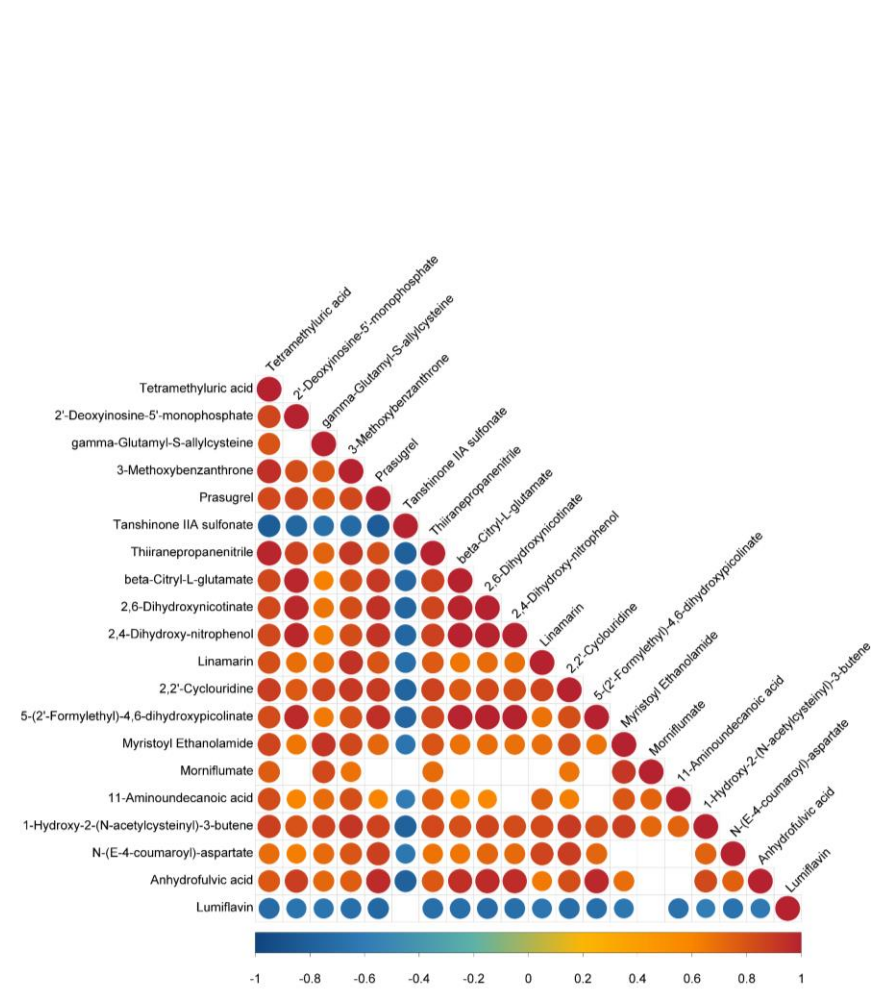

B

Yak vs Cattle\_NEG

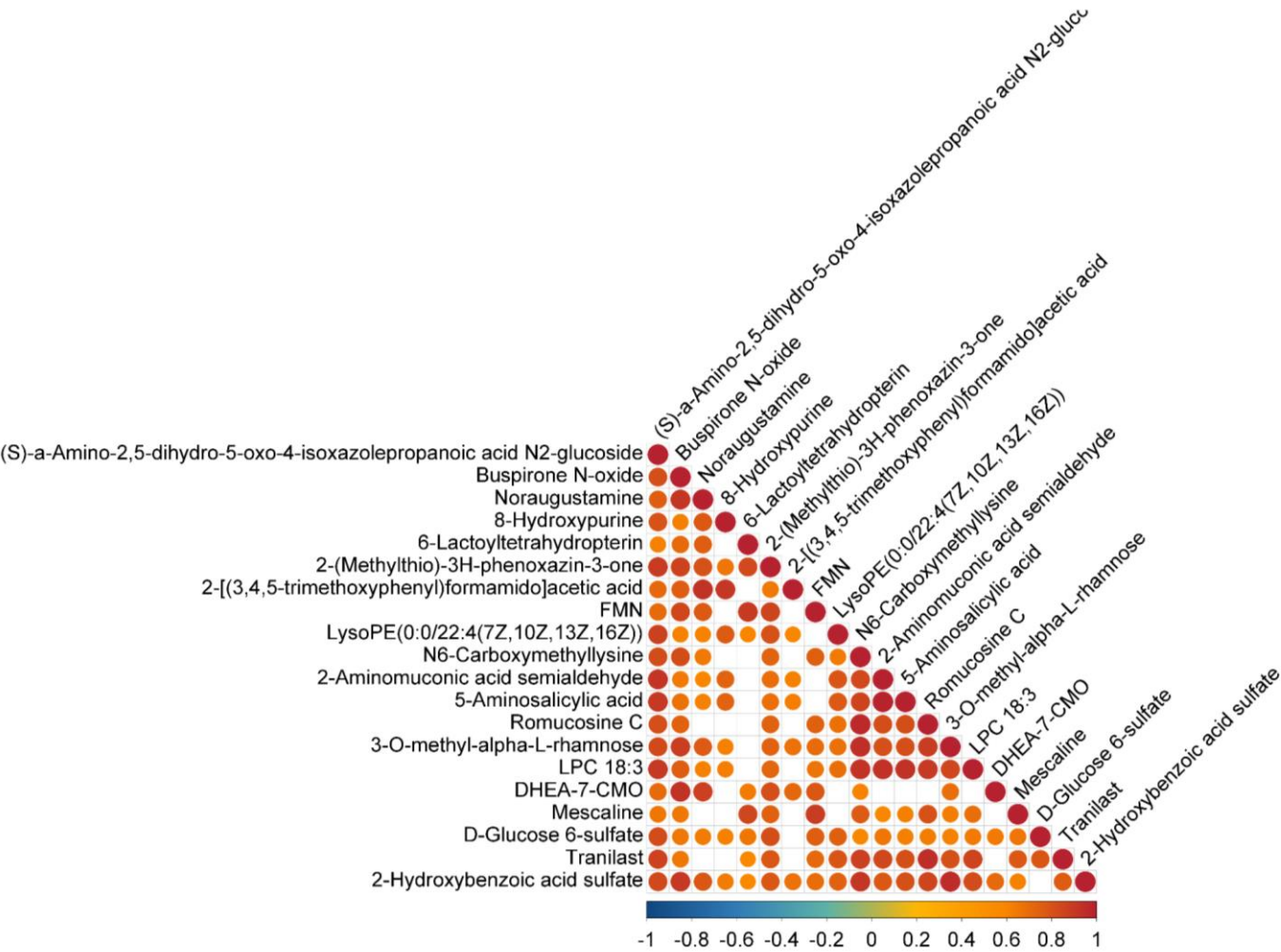

Supplement: Supplementary file 1 [file animals-15-02861-s001.zip › Figure S3.pdf]
